# Metatranscriptomic Analysis Reveals Disordered Alterations in Oropharyngeal Microbiome during the Infection and Clearance Processes of SARS-CoV-2: A Warning for Secondary Infections

**DOI:** 10.3390/biom13010006

**Published:** 2022-12-20

**Authors:** Yongzhao Zhou, Sifen Lu, Xiaozhen Wei, Ya Hu, Honghao Li, Jing Wang, Yifei Lin, Mengjiao Li, Minjin Wang, Jinmin Ma, Zhongyi Zhu, Shengying Yang, Binwu Ying, Wengeng Zhang, Bojiang Chen, Weimin Li

**Affiliations:** 1 Department of Integrated Care Management Center, Frontier Science Center of Disease Molecular Network, West China Hospital, Sichuan University, Chengdu 610041, China; 2 Precision Medicine Key Laboratory of Sichuan Province and Precision Medicine Center, West China Hospital, Sichuan University, Chengdu 610041, China; 3 Department of Anesthesiology, West China Hospital, Sichuan University, Chengdu 610041, China; 4 Center of Infectious Diseases, West China Hospital, Sichuan University, Chengdu 610041, China; 5 Department of Hospital Management, West China Hospital, Sichuan University, Chengdu 610041, China; 6 Department of Laboratory Medicine, West China Hospital, Sichuan University, Chengdu 610041, China; 7 BGI-Shenzhen & BGI-PathoGenesis Pharmaceutical Technology, Shenzhen 518083, China; 8 Department of Computer & Software, Jincheng College of Chengdu, Chengdu 611700, China

**Keywords:** metatranscriptomic sequencing, oropharyngeal microbiome, SARS-CoV-2, infection and clearance processes, secondary infections

## Abstract

This study was conducted to investigate oropharyngeal microbiota alterations during the progression of coronavirus disease 2019 (COVID-19) by analyzing these alterations during the infection and clearance processes of severe acute respiratory syndrome coronavirus 2 (SARS-CoV-2). The diagnosis of COVID-19 was confirmed by using positive SARS-CoV-2 quantitative reverse transcription polymerase chain reaction (RT-qPCR). The alterations in abundance, diversity, and potential function of the oropharyngeal microbiome were identified using metatranscriptomic sequencing analyses of oropharyngeal swab specimens from 47 patients with COVID-19 (within a week after diagnosis and within two months after recovery from COVID-19) and 40 healthy individuals. As a result, in the infection process of SARS-CoV-2, compared to the healthy individuals, the relative abundances of *Prevotella*, *Aspergillus*, and *Epstein–Barr virus* were elevated; the alpha diversity was decreased; the beta diversity was disordered; the relative abundance of Gram-negative bacteria was increased; and the relative abundance of Gram-positive bacteria was decreased. After the clearance of SARS-CoV-2, compared to the healthy individuals and patients with COVID-19, the above disordered alterations persisted in the patients who had recovered from COVID-19 and did not return to the normal level observed in the healthy individuals. Additionally, the expressions of several antibiotic resistance genes (especially multi-drug resistance, glycopeptide, and tetracycline) in the patients with COVID-19 were higher than those in the healthy individuals. After SARS-CoV-2 was cleared, the expressions of these genes in the patients who had recovered from COVID-19 were lower than those in the patients with COVID-19, and they were different from those in the healthy individuals. In conclusion, our findings provide evidence that potential secondary infections with oropharyngeal bacteria, fungi, and viruses in patients who have recovered from COVID-19 should not be ignored; this evidence also highlights the clinical significance of the oropharyngeal microbiome in the early prevention of potential secondary infections of COVID-19 and suggests that it is imperative to choose appropriate antibiotics for subsequent bacterial secondary infection in patients with COVID-19.

## 1. Introduction

The COVID-19 pandemic is a once-in-a-century public health crisis with high mortality [1], and it was caused by severe acute respiratory syndrome coronavirus 2 (SARS-CoV-2) [2]. Although it is singularly caused by SARS-CoV-2, COVID-19 can be affected by the rest of the microbiome, especially the gut. For example, a previous study confirmed that the intestinal microbial composition of patients with COVID-19 significantly changed compared with that of patients without COVID-19 [3]. Another study reported that pathogenic fungal communities, such as *Candida* and *Aspergillus*, were significantly increased in the intestinal tract of patients with COVID-19 during hospitalization [4], and another team further confirmed that the intestinal microbial community abundance of patients with COVID-19 did not return to the normal level six months after their recovery [5]. However, SARS-CoV-2 first affects the upper respiratory tract (URT), and the lungs are the most affected organ [6]. Furthermore, the main flora in the lower respiratory tract of patients with COVID-19 have been found to originate from the related flora in the URT [7], and several studies have reported that bacterial, fungal, and viral infections associated with or secondary to COVID-19 in patients deserve further attention [8]. In addition, another review described the viral–bacterial lung co-infections that occurred during the COVID-19 pandemic [9]. Thus, understanding the URT microecology of patients with COVID-19 is significant for the prevention and treatment of infections combined with COVID-19 and those secondary to it [10].

Additionally, the oropharynx is one of the components of the URT [11]. Significantly, oropharyngeal microbiome alterations could influence the severity of COVID-19 [12] and could contribute to the progression of H7N9 avian influenza [13]. However, oropharyngeal microbiota alterations in the progression of COVID-19 have not yet been thoroughly investigated.

Here, based on a metatranscriptomic sequencing analysis, we aimed to explore oropharyngeal microbiota alterations during the progression of COVID-19 by analyzing those alterations during the infection and clearance processes of SARS-CoV-2. In the infection process of SARS-CoV-2, we compared oropharyngeal microbiota alterations between healthy individuals (before infection) and patients with COVID-19 (after infection). In the clearance process of SARS-CoV-2, we compared those alterations between patients with COVID-19 (before clearance) and recovered individuals (after clearance).

## 2. Material and Methods

### 2.1. Study Design

We recruited 47 patients with COVID-19 confirmed using a positive SARS-CoV-2 quantitative reverse transcription PCR (RT-qPCR). They were diagnosed and categorized into four degrees of severity, namely, mild, moderate, severe, and critical, according to the Diagnosis and Treatment Scheme for COVID-19 released by the National Health Commission of China [14,15]. We also recruited 40 healthy controls, whose inclusion criteria included (a) being asymptomatic; (b) being negative for SARS-CoV2 determined via RT-qPCR; and (c) not being in hospital. In the infection process of SARS-CoV-2, the 40 healthy individuals represented the condition before infection, and the 47 patients with COVID-19 represented the condition after infection. In the clearance process of SARS-CoV-2, the 47 patients with COVID-19 represented the condition before clearance, and 47 patients who had recovered from COVID-19 represented the condition after clearance.

### 2.2. Patient and Public Involvement

The patients volunteered to supply their samples for this study, and they themselves were not involved in the design, the recruitment, or conduct of this study. This study was approved by the Biomedical Research Ethics Committee of West China Hospital (No. 2020 [100], No. 2020 [193], and No. 2020 [267]). Written informed consent complying with the Declaration of Helsinki was obtained from the patients or their guardians.

### 2.3. Sample Collection

During February 2020 and June 2020, we collected oropharyngeal swabs from 47 patients at two time points (within a week of COVID-19 diagnosis and two months after SARS-CoV-2 clearance) and 40 healthy individuals. To evaluate for possible contamination, six additional blank control swabs were collected.

Oropharyngeal swabs were collected according to a video of the standard maneuver published by the Chinese Society of Laboratory Medicine [16]. Briefly, a sterile flock swab was used for sampling by wiping the back wall of the pharynx with moderate force and avoiding touching the tongue. Disposable sterile gloves were used throughout the sampling process to prevent contamination. The collected samples were immediately placed in sterile tubes containing 2 mL of a viral transport medium. All collected samples were transported to the qualified SARS-CoV-2 nucleic acid testing laboratory in a mobile refrigerator and frozen at −80 °C. Relevant biohazard labels, warnings, and prompts were pasted on the transport containers and packaging materials.

### 2.4. RNA Extraction

First, the virus was inactivated in a water bath by heating to a temperature of 56 °C for 45 min. Next, 10 μL protease K solution (10 mg/mL) and 10 μL carrier RNA (1 mg/mL) were added to a sample tube (1.5 mL). Then, 200 μL or 400 μL of the sample (virus-inactivation tube storage solution) was added to the sample tube. Then, the total RNA of the 140 samples was extracted using a Concert viral RNA kit (RC1005: Concert Biotech, Xiamen, China) with an HF16 nucleic acid purification instrument according to the manufacturer’s guidelines.

### 2.5. Metatranscriptomic Sequencing

After extraction, a standard RNA-seq library was constructed using a next-generation sequencing (NGS) library construction kit (Genskey 1906, Beijing, China). Sequencing libraries of 75 bp single-end reads were generated using a NextSeq 500 High Output Kit (75 cycles) on an Illumina NextSeq 500 platform (Illumina, Inc., San Diego, CA, USA) at Genskey Gene Company (Beijing, China) with a depth of approximately 20 million reads per sample.

### 2.6. RT-qPCR Assay

Real-time RT-qPCR was performed by amplifying two target genes, open reading frame 1ab (ORF1ab) and nucleocapsid protein (N), using an RT-PCR kit (Sansure Biotech Inc., Changsha, China) with a real-time PCR thermal cycler (ABI 7500 system, Applied Biosystems instruments, Waltham, MA, USA). The primers used were as follows: SARS-CoV-2_ORF1ab-F:5′-CCCTGTGGGTTTTACACTTAA-3′, SARS-CoV-2_ORF1ab-R:5′-ACGATTGTGCATCAGCTGA-3′, SARS-CoV-2_ORF1ab-P:5′-FAM-CCGTCTGCGGTATGTGGAAAGGTTATGG-BHQ1-3′, SARS-CoV-2_N-F: 5′-GGGGAACTTCTCCTGCTAGAAT-3′, SARS-CoV-2_N-R: 5′-CAGACATTTTGCTCTCAAGCTG-3′, and SARS-CoV-2_N-P: 5′-FAM-TTGCTGCTGCTTGACAGATT-TAMRA-3′.

When ORF1ab and N genes were both positive (cycle threshold [Ct] < 37), the results were considered positive for SARS-CoV-2. When the results showed no Ct value or Ct ≥ 40 at the two detection sites, the results were considered negative for SARS-CoV-2.

### 2.7. Bioinformatics Analysis

First, software fastp (parameters: −q 15 −u 40 −l 50, version:0.19.5) [17] was used to filter low-quality reads and remove adapters; Komplexity (parameters: −t 0.4, version: November 2019) [18] was used to remove low-complexity reads from the raw data. The filtered reads were then mapped to the Ensembl 84 (GRCh38) human reference genome in order to remove human sequences using HISAT2 (version 2.1.0) with default parameters [19]. The read counts before and after human transcript alignment are shown in the Supplementary File (Table S4). Next, the unmapped reads were annotated with taxonomic classifications using Kraken2 (version 2.0.9, parameters: −threads 24 −confidence 0.1) [20] with a self-built database (built by downloading all the complete genomes from the NCBI Refseq database, including the SARS-CoV-2 reference NC_045512.2. Only the genomes of archaea, bacteria, fungi, and viruses were selected for building a classification database for Kraken2 (k = 35, ℓ = 31)). Finally, all microorganisms satisfying all the following criteria were retained for subsequent analyses: (1) archaea, bacteria, fungi, or viruses; (2) 10-fold higher filtered reads per million than that in the negative control; (3) no batch effect; (4) no known contamination; and (5) only human viruses. Gram-negative and gram-positive bacteria were predicted by using the Microbe Directory [21]. The analysis of antibiotic resistance genes (ARGs) was carried out as follows: First, unmapped reads were assemblied using SPAdes (version: 3.13.0) with default parameters [22], and scaffolds < 150 bp were removed. Then, MetaGeneMark [23] was used to predict protein coding genes from the above filtered scaffolds. Next, the abundances of ARGs were annotated by aligning the sequences of the above predicted genes to the Comprehensive Antibiotic Resistance Database (CARD 2020; https://card. mcmaster.ca (accessed on 15 September 2020)) [24] using the HISAT2 alignment method with default parameters. Each gene was filtered to have at least two matching reads. Finally, ARGs only occurred in >25% of the samples, which were used for further statistical analyses.

### 2.8. Statistical Analyses

Statistical analyses were performed using R software (version 3.5.1). The relative abundance (the abundance of a particular genus in each sample divided by the sum of the abundance of all genera in that sample) was used to quantify the abundance of the oropharyngeal microbiome. Alpha diversity was analyzed using the vegan function in R package. The principal coordinate analysis (PCoA) was based on Bray–Curtis dissimilarity (weighed) distance matrices [25]. The differences in the alpha diversity between the groups were evaluated using the Mann–Whitney (Kruskal–Wallis) test. An analysis of similarities (ANOSIM) was used to determine significant differences between two or more groups of sampling units [26]. The relative abundances of the oropharyngeal microbiome between different groups were calculated using the Wilcoxon rank-sum test. *p*-values of < 0.05 *, < 0.01 **, and < 0.001 *** were considered statistically significant. We provided the code for statistics and plot in Gitee (https://gitee.com/zhengtang/biomolecules/releases/tag/Release).

## 3. Results

### 3.1. Study Cohort

The information of the 47 patients with COVID-19 (period of disease group: PDG, and convalescent group: CG) and the 40 healthy individuals (healthy control group: HCG) is shown in Table 1.

### 3.2. Relative Abundance Alterations in Oropharyngeal Bacteria during the Infection and Clearance Processes of SARS-CoV-2

**Figure 1:**
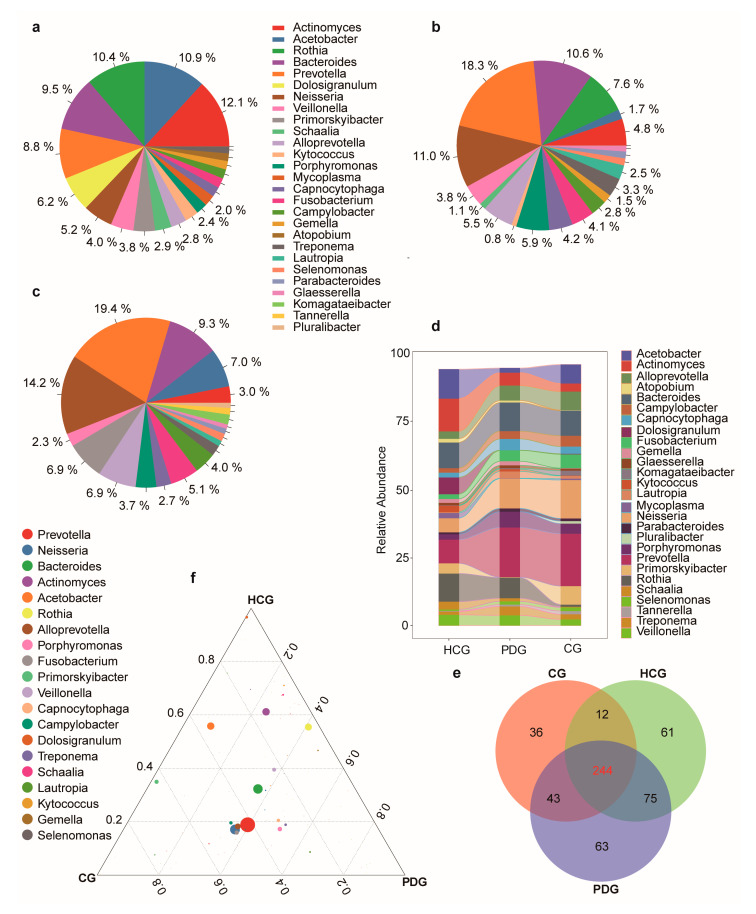
Oropharyngeal bacterial alterations among HCG, PDG, and CG. Average compositions of relative abundance of the top 20 bacterial genera for (**a**) HCG, (**b**) PDG, and (**c**) CG. (**d**) Alterations in the top 20 bacterial genera among the three groups. (**e**) The shared and unique genera of the three groups. (**f**) Ternary plots depicting the 244 shared genera of the three groups. The sum of the proportion for one specific bacterium in the three groups was set to 1, and the proportion of one specific bacterium in each group is equal to its corresponding relative abundance divided by the relative abundance sum of these bacteria in the three groups. The sizes of the circles represent the relative abundance of the genus. HCG: healthy control group. PDG: period of disease group. CG: convalescent group.

Overall, 534 genera of bacteria were detected in the 134 study subjects. The relative abundances of the top 20 bacterial profiles in the HCG, PDG, and CG are shown in Figure 1a–c. Consistent with previous studies [27], the major bacterial genera in the HCG were *Actinomyces*, *Acetobacter*, *Rothia*, *Bacteroides*, and *Prevotella* (Figure 1a). The major bacterial genus in the PDG was *Prevotella*, and the relative abundance of *Prevotella* in the PDG was significantly higher than that in the HCG (Figure 1a,b,d; Supplementary Table S1; *p* < 0.05). After SARS-CoV-2 was cleared, the relative abundance of *Prevotella* in the CG was also significantly higher than that in the HCG (Figure 1a,c,d; Supplementary Table S1; *p* < 0.05), and it was higher than that in the PDG (Figure 1b–d; Supplementary Table S1; *p* > 0.05). Among the 534 detected genera, 244 were found in all three groups; 63 were only detected in the PDG, 36 were only detected in the CG, and 61 were only detected in the HCG (Figure 1e). Among the 244 shared genera, *Prevotella* was the dominant genus in the PDG and CG (Figure 1f). The total number/abundance of the 534 genera of bacteria is shown in the Supplementary File (Table S5).

### 3.3. Relative Abundance Alterations in Oropharyngeal Fungi during the Infection and Clearance Processes of SARS-CoV-2

**Figure 2:**
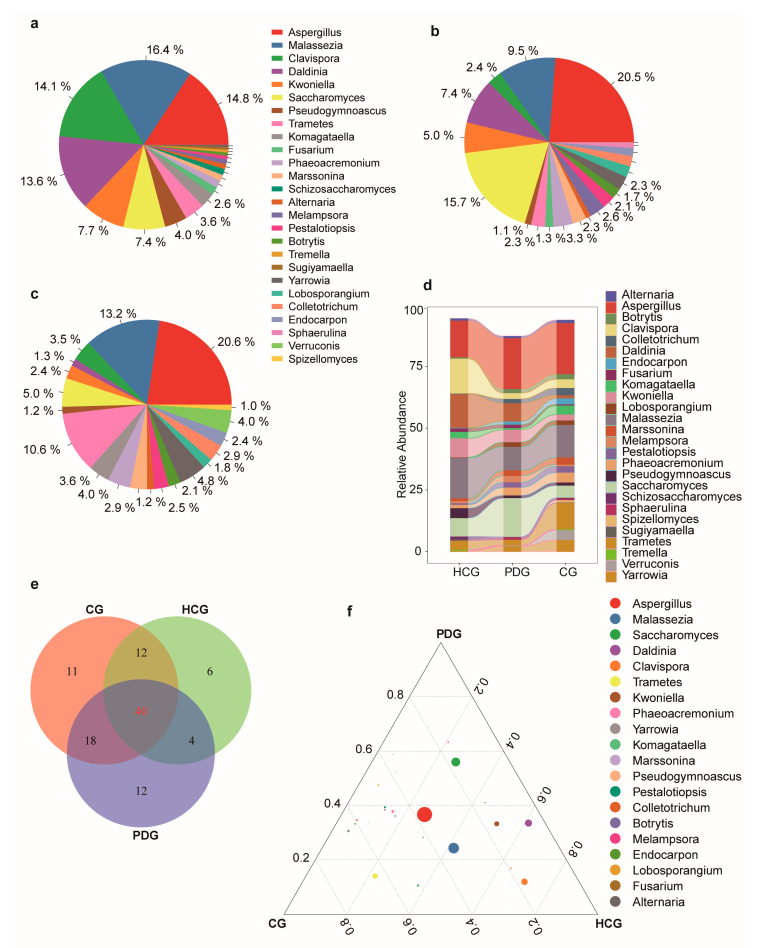
Oropharyngeal fungal alterations among HCG, PDG and CG. Average compositions of relative abundance of the top 20 fungal genera for (**a**) HCG, (**b**) PDG, (**c**) CG. (**d**) Alterations in the top 20 fungal genera among the three groups. (**e**) The shared and unique genera of the three groups. (**f**) Ternary plots depicting the 244 shared fungal genera of the three groups. The sum of the proportion for one specific fungus in the three groups was set to 1, and the proportion of one specific fungus in each group is equal to its corresponding relative abundance divided by the relative abundance sum of this fungus in the three groups. The sizes of the circles represent the relative abundance of the genus. HCG: healthy control group. PDG: period of disease group. CG: convalescent group.

Overall, 103 genera of fungi were detected in the 134 study subjects. The relative abundances of the top 20 fungal profiles in the HCG, PDG, and CG are shown in Figure 2a–c. The major genera in the PDG was *Aspergillus*, and the relative abundance of *Aspergillus* in the PDG was significantly higher than that in the HCG (Figure 2a,b,d; Supplementary Table S2; *p* < 0.05). After SARS-CoV-2 was cleared, the relative abundance of *Aspergillus* in the CG was also significantly higher than that in the HCG (Figure 2a,c,d; Supplementary Table S2; *p* < 0.05), and it was higher than that in the PDG (Figure 2b–d; Supplementary Table S2; *p* > 0.05). Among the 103 detected genera, 40 were found in all three groups; 12 were only detected in the PDG, 11 were only detected in the CG, and 6 were only detected in the HCG (Figure 2e). Among the 40 shared genera, *Aspergillus* was the dominant genus in the PDG and CG (Figure 2f).

### 3.4. Relative Abundance Alterations in Oropharyngeal Viruses during the Infection and Clearance Processes of SARS-CoV-2

**Figure 3:**
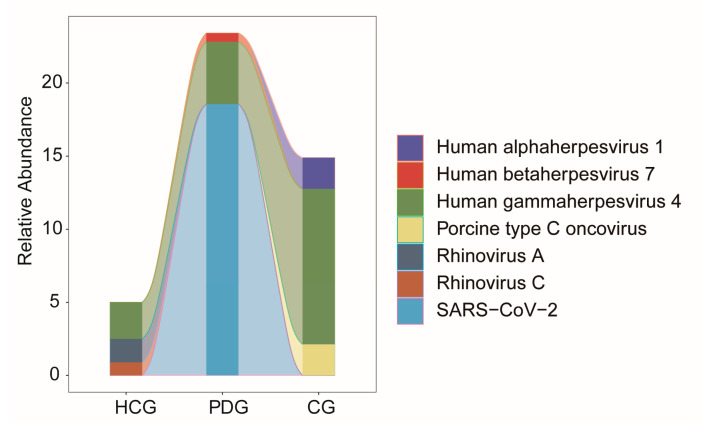
Oropharyngeal viral alterations among HCG, PDG and CG. The relative abundance changes of 7 oropharyngeal viral species (including SARS-CoV-2) among HCG, PDG and CG. HCG: healthy control group. PDG: period of disease group. CG: convalescent group.

Overall, *Human gamma herpesvirus 4* (also known as *Epstein–Barr virus* (EBV)), *Rhinovirus A*, and *Rhinovirus C* were the major viruses in the HCG (Figure 3). SARS-CoV-2 infection mainly co-occurred with EBV, followed by *Human beta herpesvirus 7* (HHV-7), in the PDG (Figure 3). After SARS-CoV-2 was cleared, the relative abundance of EBV was higher in the CG than in the HCG or the PDG (Figure 3; Supplementary Table S3; *p* > 0.05).

### 3.5. Diversity Alterations in Oropharyngeal Microbiome during the Infection and Clearance Processes of SARS-CoV-2

**Figure 4:**
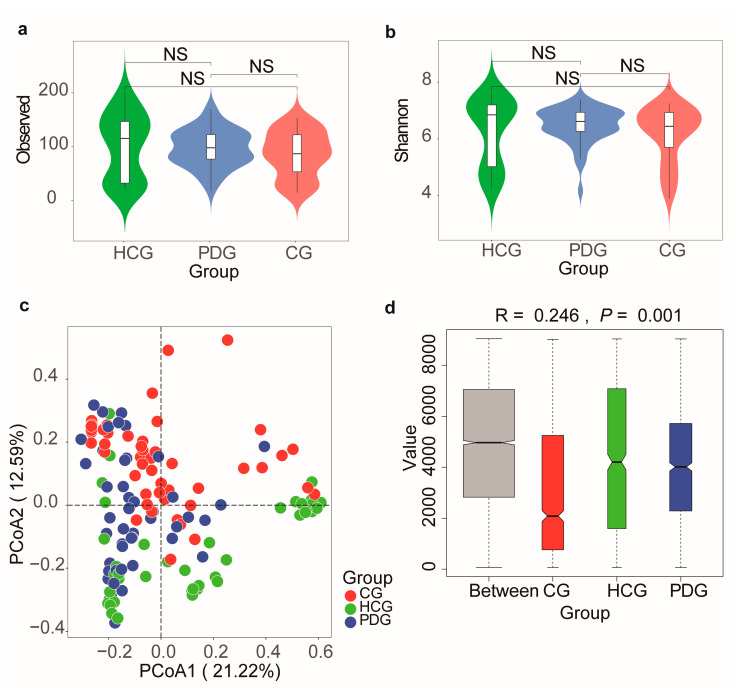
Oropharynx microbial alterations in diversities among HCG, PDG, and CG. Alpha diversity among HCG, PDG, and CG, as illustrated by the (**a**) Observed index (NS: *p* > 0.05) and (**b**) Shannon index (NS: *p* > 0.05). (**c**) Principal coordinate analysis (PCoA) based on Bray–Curtis (weighed) distance showed obvious changes in beta diversity among HCG, PDG, and CG. The colors represent three different groups. PCoA1 and PCoA2 represent the top two principal coordinates that captured most of the diversity. The fraction of diversity captured by the coordinate is valued as a percentage. (**d**) The analysis of similarities (ANOSIM) based on Bray–Curtis (weighed) distance (R > 0, and *p* < 0.05). The R means the statistical value of ANOSIM, and y-axis value means Bray–Curtis rank. NS: not significant. HCG: healthy control group. PDG: period of disease group. CG: convalescent group.

Then, we analyzed the alterations in the alpha and beta diversities among the HCG, PDG, and CG. Consequently, the PDG and CG showed a lower, but non-significant, alpha diversity than the HCG, as illustrated by the Observed index, Shannon index (Figure 4a,b; *p* > 0.05), Chao1 index, and Simpson index (Supplementary Figure S1a,b; *p* > 0.05). The result of beta diversity showed that the PDG was slightly separate from the HCG and that the CG was slightly separate from the HCG and the PDG (Figure 4c). The ANOSIM plot showed that the intergroup difference was greater than the intra-group difference (Figure 4d, R = 0.246, *p* = 0.001).

### 3.6. Potential Function Alterations in Oropharyngeal Microbiome during the Infection and Clearance Processes of SARS-CoV-2

**Figure 5:**
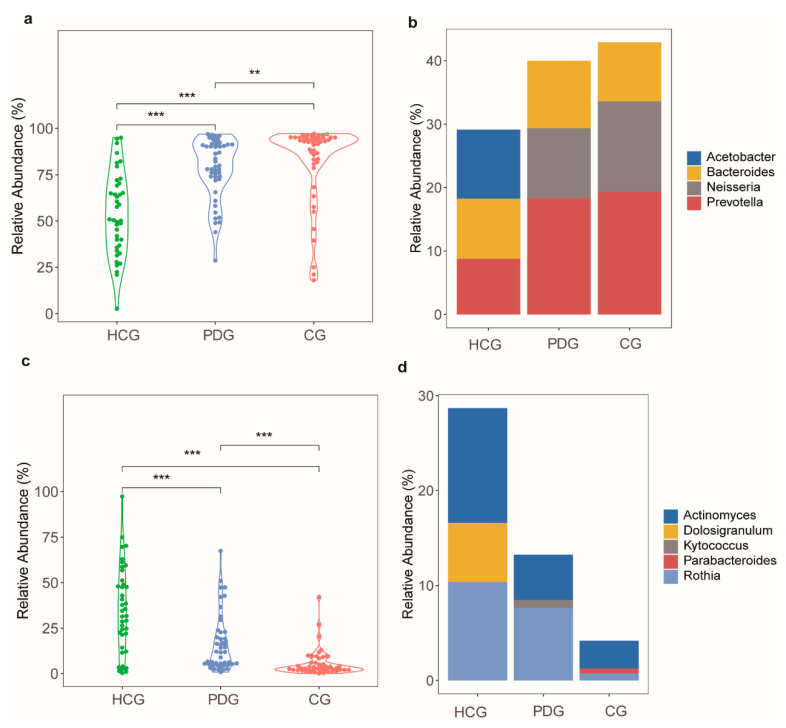
Alterations in Gram-negative and Gram-positive bacteria among HCG, PDG, and CG. (**a**) The relative abundances of Gram-negative bacteria in PDG and in CG were significantly greater than that in HCG, (**b**) and the major bacterium contributing to this was *Prevotella*. (**c**) The relative abundance of Gram-positive bacteria in PDG and in CG was significantly smaller than that in HCG, (**d**) and the major bacteria contributing to this were *Rothia* and *Actinomyces*. The *p*-values were confirmed by the Wilcoxon rank-sum test. HCG: healthy control group. PDG: period of disease group. CG: convalescent group. *p*-values of < 0.01**, and < 0.001*** were considered statistically significant.

Furthermore, to explore the potential function alterations, we compared the relative abundances of Gram-negative and Gram-positive bacteria among the HCG, PDG, and CG. Interestingly, the relative abundance of the Gram-negative bacteria in the PDG was significantly higher than that in the HCG (Figure 5a; *p* < 0.001). After SARS-CoV-2 was cleared, the relative abundance of the Gram-negative bacteria in the CG was also significantly higher than that in the HCG and PDG (Figure 5a; *p* < 0.01), and the major bacterium contributing to this was *Prevotella* (Figure 5b). The relative abundance of the Gram-positive bacteria in the PDG was significantly lower than that in the HCG (Figure 5c; *p* < 0.001). After SARS-CoV-2 was cleared, the relative abundance of the Gram-positive bacteria in the CG was also significantly lower than that in the HCG and PDG (Figure 5c; *p* < 0.001), and the major bacteria contributing to this were *Rothia* and *Actinomyces* (Figure 5d).

**Figure 6:**
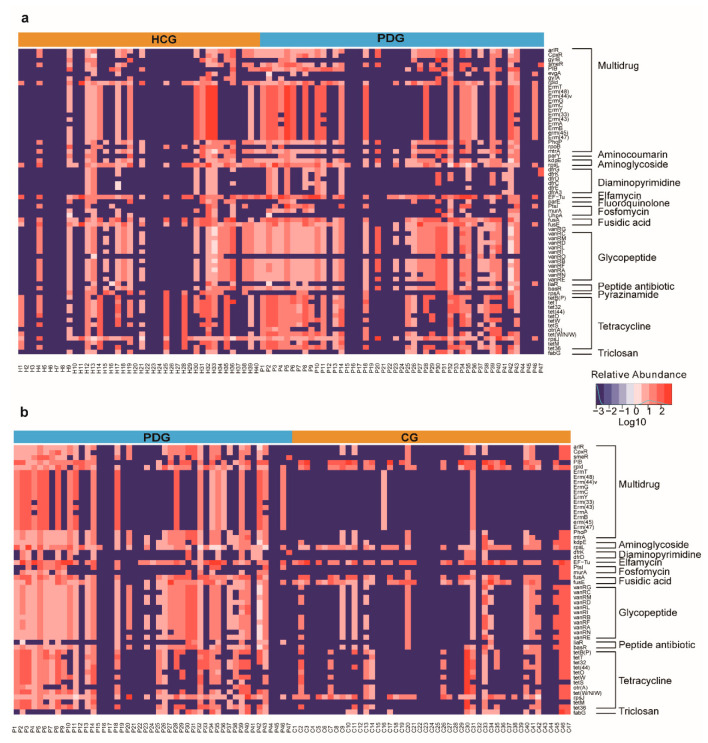
Alterations in antibiotic resistance genes among HCG, PDG, and CG. (**a**) Heatmap for the expressions of microbial antibiotic resistance genes between HCG and PDG. (**b**) Heatmap for the expressions of microbial antibiotic resistance genes between PDG and CG. The relative abundance changes from low to high are indicated by color changes from blue to red. The log10 value was used to show the abundance of antibiotic resistance gene. HCG: healthy control group. PDG: period of disease group. CG: convalescent group.

Importantly, we also analyzed the alterations in ARGs among the HCG, PDG, and CG. Consequently, we identified genes that confer resistance to 13 classes of antibiotics, with variations across the HCG, PDG, and CG (Figure 6a,b). The expressions of several ARGs (especially multi-drug resistance, glycopeptide, and tetracycline) in the PDG were higher than those in the HCG. After SARS-CoV-2 was cleared, the expressions of these genes in the CG were lower than those in the PDG, and they were different from those in the HCG. A statistical analysis of the ARGs is shown in the Supplementary File (Tables S6 and S7).

## 4. Discussion and Conclusions

This study is the first to use a metatranscriptomic sequencing analysis to show alterations in the abundance, diversity, and potential function of the oropharyngeal microbiome during both the infection and clearance processes of SARS-CoV-2 in 47 patients with COVID-19, 47 recovered patients, and 40 healthy individuals.

Interestingly, we found that the relative abundance of *Prevotella* in the PDG was significantly higher than that in the HCG, indicating that SARS-CoV-2 infection is related to *Prevotella* perturbation, which is consistent with previous findings showing that *Prevotella* was the main bacterium in the URT of patients with COVID-19 [28]. The relative abundance of *Prevotella* in the CG was also higher than that in the HCG and PDG, indicating that *Prevotella* dysbiosis persisted after SARS-CoV-2 clearance in the patients who had recovered from COVID-19, suggesting that exposure to SARS-CoV-2 infection may have a long-term effect on the alterations in oropharyngeal bacteria. This is possibly because the oral cavity is one of the first entry points into the body, and oral pathogens in the lungs can cause pulmonary co-infections; respiratory viral infections increase susceptibility to secondary bacterial infections of the lungs [29,30]. Thus, these findings indicate that attention should be paid to secondary bacterial infections in patients who have recovered from COVID-19, especially those caused by *Prevotella*.

Additionally, the relative abundance of *Aspergillus* in the PDG was significantly higher than that in the HCG, indicating that SARS-CoV-2 infection is associated with *Aspergillus* perturbation, which is consistent with previous findings reporting that some patients with COVID-19 hospitalized for acute respiratory distress syndrome have co-infections with *Aspergillus* [31]. The relative abundance of *Aspergillus* in the CG was also higher than that in the HCG and PDG, indicating that *Aspergillus* dysbiosis persisted after SARS-CoV-2 clearance in the patients who had recovered from COVID-19, suggesting that exposure to SARS-CoV-2 infection may also have a long-term effect on the alterations in oropharyngeal fungi. Moreover, *Aspergillus fumigatus* is the most common microorganism that has been identified to cause secondary fungal pulmonary infections in patients with COVID-19 [32], and this is probably because the immune response in some patients with COVID-19 is dysfunctional, leaving them vulnerable to secondary lung infections. Thus, these findings indicate that attention should be paid to secondary fungal infections in patients who have recovered from COVID-19, especially those caused by *Aspergillus*.

Furthermore, the relative abundance of EBV in the PDG was higher than that in the HCG, indicating that SARS-CoV-2 infection is associated with EBV elevation. A previous study reported that the most common virus in the URT of patients with COVID-19 was EBV, thus supporting our findings [28]. The relative abundance of EBV in the CG was also higher than that in the HCG and PDG, indicating that EBV elevation persisted despite SARS-CoV-2 clearance in the patients who had recovered from COVID-19, suggesting that exposure to SARS-CoV-2 infection may also have a long-term effect on the alterations in oropharyngeal viruses. Moreover, EBV is a common virus that has been identified to cause secondary virus infections in patients with COVID-19 [33]. Thus, these findings indicate that attention should be paid to secondary viral infections in patients who have recovered from COVID-19, especially those caused by EBV.

Next, the PDG and CG showed a lower, but non-significant, alpha diversity than the HCG, as illustrated by the Observed index, Shannon index Chao1 index, and Simpson index, conforming to previous findings showing that there were no significant differences in alpha diversity between patients with COVID-19 and healthy controls [34]. The PCoA results for beta diversity showed that the PDG was slightly separate from the HCG and that the CG was slightly separate from the HCG and PDG, which is partly conforming to the findings of a previous report revealing that the microbial community distribution in recovered patients was significantly different from that in patients with COVID-19 and healthy individuals by using a 16S rRNA sequencing analysis [35]. This is probably because exposure to SARS-CoV-2 may have a long-term effect on the microbial community distribution of the oropharyngeal microbiome.

Finally, the relative abundance of the Gram-negative bacteria in the PDG was significantly higher than that in the HCG, indicating that SARS-CoV-2 infection may perturb the composition of bacteria, which is consistent with previous findings showing that Gram-negative pathogens are the major cause of bacterial pneumonia in patients critically ill with COVID-19 [36]. The relative abundance of the Gram-negative bacteria in the CG was also significantly higher than that in the HCG and PDG, indicating that this impact persisted after SARS-CoV-2 clearance in the patients who had recovered from COVID-19; this is concerning because it may affect the risk of subsequent infections [37]. It is remarkable that Gram-negative bacteria are one of the most serious global public health problems because of their high resistance to antibiotics [38]; patients hospitalized for COVID-19 often use broad-spectrum antimicrobials, which may contribute to the development of antimicrobial resistance [39]. Thus, we analyzed the alterations in ARGs. Consequently, the expressions of several ARGs (especially multi-drug resistance, glycopeptide, and tetracycline) in the PDG were higher than those in the HCG, indicating that SARS-CoV-2 infection may perturb the abundance of resistance genes. The expressions of these resistance genes in the CG were lower than those in the PDG, and they were different from those in the HCG, indicating that the drug resistance genes of the patients who had recovered from COVID-19 did not return to the normal state observed in healthy individuals after SARS-CoV-2 clearance. Although further research is warranted to better understand the landscape of antimicrobial resistance during COVID-19, such as quantifying the true rate of antibiotic resistance to inform the use of appropriate empirical therapies and to understand the types of resistant co-infections and secondary infections of patients with COVID-19 [39], our analysis of bacterial ARGs of the oropharyngeal microbiome in the URT of patients with COVID-19 might help to better choose the appropriate antibiotics for subsequent bacterial secondary infections in patients with COVID-19.

However, this study had some limitations. First, in the infection process of SARS-CoV-2, individual differences in the impact of oropharyngeal microbiome alterations were not considered because healthy samples of the patients confirmed to have COVID-19 before infection were largely unavailable. Second, the major impact of antibiotics on the respiratory tract flora in the infection and clearance processes of SARS-CoV-2 was not analyzed and deserves further study. Third, follow-up data on the secondary infections of the patients who had recovered from COVID-19 are lacking. Fourth, only the genus-level abundance was shown, no other taxonomic classification abundance. In the future, we will conduct further research to confirm the mechanism of the oropharyngeal microbiome effect on the development of COVID-19.

In summary, we revealed the alterations in abundance, diversity, and potential function of the oropharyngeal microbiome in the infection and clearance processes of SARS-CoV-2 for the first time using metatranscriptomic sequencing data. In the infection process of SARS-CoV-2, compared to the HCG, the relative abundances of *Prevotella*, *Aspergillus*, and EBV were elevated; the alpha diversity was decreased; the beta diversity was disordered; the relative abundance of Gram-negative bacteria was increased; and the relative abundance of Gram-positive bacteria was decreased. After the clearance of SARS-CoV-2, compared to the HCG and PDG, the above disordered alterations persisted in the patients who had recovered from COVID-19 and did not return to the normal level observed in healthy individuals. Additionally, the expression of several antibiotic resistance genes (especially multi-drug resistance, glycopeptide, and tetracycline) in the PDG were higher than those in the HCG. After SARS-CoV-2 was cleared, the expressions of these genes in the CG were lower than those in the PDG, and they were different from those in the HCG.

In conclusion, we provided evidence that warns of potential secondary infections with oropharyngeal bacteria, fungi, and viruses in patients who have recovered from COVID-19; this evidence also highlights the clinical significance of the oropharyngeal microbiome in the early prevention of potential secondary infections in COVID-19 and suggests that it is necessary to choose appropriate antibiotics for subsequent bacterial secondary infections in patients with COVID-19.

## Figures and Tables

**Table 1:** Demographics and clinical characteristics of the participants.

Characteristics	Patients with COVID-19	Healthy Controls
Number of subjects	47	40
Age-years (mean ± SD)	44.7 ± 15.3	45.8 ± 16.4
Gender		
Male	20/47(42.5%)	20/40(50.0%)
Female	27/47(57.5%)	20/40(50.0%)
Disease severity category		
Mild	5/47 (10.6%)	NA
Moderate	25/47 (53.2%)	NA
Severe	10/47 (21.3%)	NA
Critical	7/47 (14.9%)	NA
Symptoms at admission		
Fever	29/47 (61.7%)	NA
Cough	29/47 (61.7%)	NA
Sputum	19/47 (40.4%)	NA
Sore throat	7/47 (14.9%)	NA
Shortness of breath	17/47 (36.2%)	NA
Received antibiotics during first week of hospitalization by disease severity ^1^		
Mild	1/47 (2.1%)	NA
Moderate	3/47 (6.4%)	NA
Severe	5/47 (10.6%)	NA
Critical	7/47 (14.9%)	NA
Received antivirals during first week of hospitalization by disease severity ^2^		
Mild	4/47 (8.5%)	NA
Moderate	24/47 (51.1%)	NA
Severe	10/47 (21.3%)	NA
Critical	7/47 (14.9%)	NA
Comorbidities		
Hypertension	7/47 (14.9%)	2/40 (5.0%)
Heart disease	4/47 (8.5%)	0
Diabetes	5/47 (10.6%)	2/40 (5.0%)
Chronic obstructive pulmonary disease	1/47 (2.1%)	1/40 (2.5 %)
Chronic bronchitis	2/47 (4.3%)	0

^1^ The antibiotics included quinolones and cephalosporins. ^2^ The antivirals included lopinavir/ritonavir, ribavirin, oseltamivir, and interferon.

## Data Availability

The sequencing data from this study were deposited in the CNSA (https://db.cngb.org/cnsa/) of CNGBdb with accession number CNP0001393.

## Supplementary Materials

The following supporting information can be downloaded at: https://www.mdpi.com/article/10.3390/biom13010006/s1, Figure S1: Alpha diversity among PDG, CG and HCG;Table S1:The P-values of bacteria among HCG, PDG and CG; Table S2:The P-values of fungi among HCG, PDG and CG; Table S3: The P-values of vi-ruses among HCG, PDG and CG; Table S4: The read counts of the 140 involved sample; Table S5: The total abundance of the 534 genera of bacteria; Table S6: Statistical analysis of antibiotic re-sistance gene abundance in Figure 6a; Table S7: Statistical analysis of antibiotic resistance gene abundance in Figure 6b.
