# Supplementary material for: Metatranscriptomic Analysis Reveals Disordered Alterations in Oropharyngeal Microbiome during the Infection and Clearance Processes of SARS-CoV-2: A Warning for Secondary Infections
Source: Biomolecules. 2022 Dec 20;13(1):6. doi: 10.3390/biom13010006 (PMC9856197; doi:10.3390/biom13010006)
Supplement: Supplementary file 1 [file biomolecules-13-00006-s001.zip › Supplementary Material.pdf]

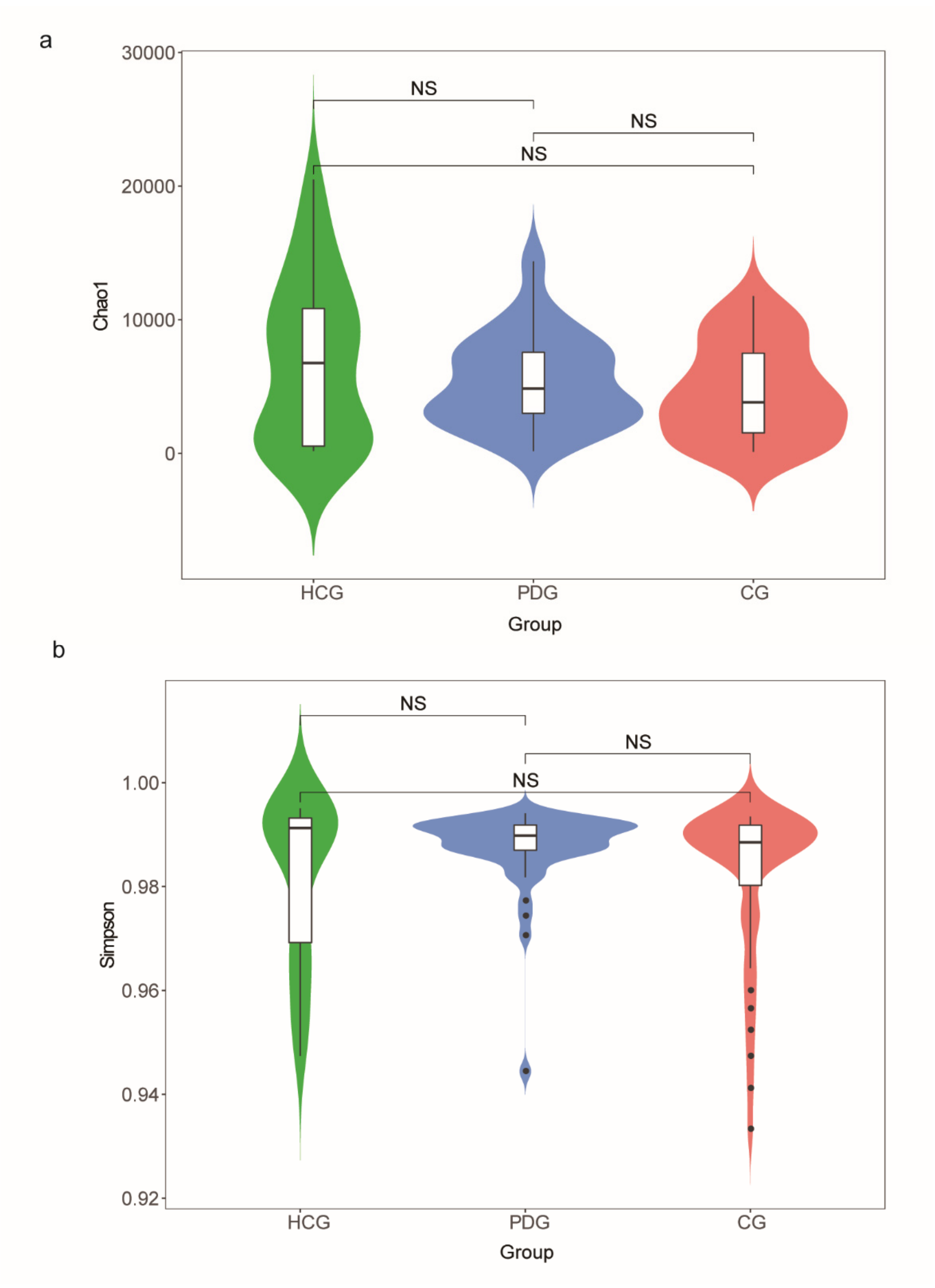

Figure S1. There were little changes in alpha diversity among PDG, CG and HCG, including (**a**) Chao1 index ($P > 0.05$), (**b**) Simpson index ($P > 0.05$).

Table S1. The *P*-values of bacteria among HCG, PDG and CG.

Genera	HCG VS PDG	HCG VS CG	PDG VS CG
Acetobacter	0.5523845	0.08171029	0.0006542014
Actinomyces	0.2349385	0.0009957278	0.0003088818
Alloprevotella	0.008206548	0.016134	0.6422
Atopobium	0.02341573	0.0004587798	0.007678531
Bacteroides	0.9101219	0.5037377	0.5827475
Campylobacter	0.02676588	0.02904887	0.7960784
Capnocytophaga	0.006395767	0.2188402	0.2481401
Dolosigranulum	0.000001640764	0.00000001263839	0.1644374
Fusobacterium	0.01536983	0.002327948	0.2626943
Gemella	0.2613953	0.0008773248	0.05959559
Glaesserella	0.08602148	0.3379769	0.4714011
Komagataeibacter	0.713456	0.2517327	0.4948042
Kytococcus	0.001744615	0.00000998748	0.009237343
Lautropia	0.005378676	0.06210935	0.3961506
Mycoplasma	0.0006483429	0.0004287703	0.7688339
Neisseria	0.05095998	0.009386655	0.1999062
Parabacteroides	0.03678525	0.2889619	0.3276759
Pluralibacter	NA	0.2887304	0.2887304
Porphyromonas	0.0009080425	0.06887038	0.2698042
Prevotella	0.00001108758	0.001887111	0.6486412

Primorskyibacter	0.428352	0.002437779	0.000002316134
Rothia	0.1070962	0.00001601009	0.00003950644
Schaalia	0.00594972	0.00003496756	0.0003304191
Selenomonas	0.004095867	0.04803979	0.4454585
Tannerella	0.0003585079	0.003201938	0.9762069
Treponema	0.0007500733	0.1022729	0.09728571
Veillonella	0.9336206	0.1158651	0.01330685

Table S2. The *P*-values of fungi among HCG, PDG and CG.

Genera	HCG VS PDG	HCG VS CG	PDG VS CG
Alternaria	0.7559474	0.6071313	0.8426529
Aspergillus	0.02015608	0.003807618	0.6063145
Botrytis	0.0007215124	0.00003254014	0.4486583
Clavispora	0.002421023	0.1218477	0.07537623
Colletotrichum	0.2475572	0.002099082	0.06996017
Daldinia	0.1177105	0.1002397	1
Endocarpon	0.00004011484	0.0000001235903	0.1312304
Fusarium	0.05465945	0.249943	0.3765705
Komagataella	0.2720938	0.01645642	0.0006122702
Kwoniella	0.3009716	0.7583863	0.4755281
Lobosporangium	0.001016808	0.0005776653	0.9751362

Malassezia	0.2967947	0.3963373	0.07579827
Marssonina	0.001642283	0.00003318598	0.2702668
Melampsora	0.2351563	0.3127587	0.8549763
Pestalotiopsis	0.0001137928	0.00007326745	0.8398866
Phaeoacremonium	0.0007765106	0.000005760552	0.3704947
Pseudogymnoascus	0.006313764	0.02274122	0.6108436
Saccharomyces	0.8860856	0.4767524	0.4467737
Schizosaccharomyces	0.02717674	0.01229319	0.6723804
Sphaerulina	0.001100909	0.01408942	0.3053589
Spizellomyces	0.3277167	0.4587604	0.1271456
Sugiyamaella	0.004080605	0.04008294	0.4587604
Trametes	0.1474609	0.001213871	0.00005219259
Tremella	0.01015853	0.1189486	0.2996268
Verruconis	0.01216164	0.0000000083559	0.00006941079
Yarrowia	0.02344477	0.0000003240681	0.001809771

Table S3. The *P*-values of viruses among HCG, PDG and CG.

Genera	HCG VS PDG	HCG VS CG	PDG VS CG
Human alphaherpesvirus 1	NA	0.2887304	0.2887304
Human betaherpesvirus 7	0.327716665	NA	0.368403472
Human gammaherpesvirus 4	0.314552254	0.1204879	0.544812611
Porcine type-C oncovirus	0.327716665	NA	0.368403472
Rhinovirus A	0.327716665	0.3684035	NA
Rhinovirus C	0.327716665	0.3684035	NA
SARS-CoV-2	0.001753917	NA	0.003776169

Table S4. The read counts of the 140 involved sample.

Samples	total_clean_reads	human_map_reads	unmap.reads
PG1	20,311,598	159,805	20,151,793
PG10	18,263,705	9,632,479	8,631,226
PG11	21,628,608	483,489	21,145,119
PG12	15,184,163	8,162,043	7,022,120
PG13	20,723,215	1,560,350	19,162,865
PG14	21,600,723	2,781,588	18,819,135
PG15	23,431,187	19,808,427	3,622,760
PG16	13,604,539	11,443,621	2,160,918
PG17	18,599,509	17,367,801	1,231,708
PG18	19,574,130	8,076,837	11,497,293
PG19	17,766,250	17,092,809	673,441
PG2	17,347,841	1,787,154	15,560,687
PG20	16,884,651	11,614,877	5,269,774
PG21	16,429,065	13,898,476	2,530,589
PG22	22,463,019	18,904,535	3,558,484
PG23	43,239,914	23,267,071	19,972,843
PG24	25,814,754	18,458,003	7,356,751
PG25	38,150,343	8,724,193	29,426,150
PG26	25,005,348	1,819,497	23,185,851
PG27	17,433,507	6,602,780	10,830,727
PG28	17,357,136	12,990,649	4,366,487

PG29	15,432,730	8,271,801	7,160,929
PG3	16,092,928	1,511,923	14,581,005
PG30	19,819,669	14,792,613	5,027,056
PG31	17,427,264	12,810,666	4,616,598
PG32	19,883,209	1,803,954	18,079,255
PG33	13,934,611	9,589,474	4,345,137
PG34	17,405,966	1,028,588	16,377,378
PG35	21,259,229	4,067,677	17,191,552
PG36	12,913,770	3,498,684	9,415,086
PG37	18,602,635	2,934	18,599,701
PG38	21,629,939	5,802,530	15,827,409
PG39	18,816,035	2,819,379	15,996,656
PG4	17,646,712	159,470	17,487,242
PG40	16,626,344	6,774,929	9,851,415
PG41	19,134,947	14,928,081	4,206,866
PG42	18,523,131	462,098	18,061,033
PG43	18,941,024	1,314,201	17,626,823
PG44	20,144,153	18,294,540	1,849,613
PG45	17,788,964	15,929,747	1,859,217
PG46	16,274,390	9,540,829	6,733,561
PG47	16,341,213	15,263,252	1,077,961
PG5	12,578,301	2,643,753	9,934,548
PG6	19,144,344	1,974,298	17,170,046
PG7	20,487,328	1,414,652	19,072,676
PG8	13,918,568	575,946	13,342,622
PG9	17,240,982	3,311,853	13,929,129
CG18	31,544,214	26,236,498	5,307,716
CG12	26,503,858	24,422,041	2,081,817
CG28	22,756,203	17,231,721	5,524,482
CG43	31,845,280	28,640,172	3,205,108
CG16	31,469,900	21,576,462	9,893,438
CG37	24,466,904	17,754,987	6,711,917
CG10	28,900,665	12,389,926	16,510,739
CG27	18,721,455	14,901,068	3,820,387
CG33	22,089,222	2,179,317	19,909,905
CG44	19,157,780	17,442,794	1,714,986
CG23	14,350,242	13,375,692	974,550
CG24	14,889,680	13,826,313	1,063,367
CG45	15,738,371	12,642,136	3,096,235
CG46	16,225,280	7,223,812	9,001,468
CG47	20,378,645	10,857,522	9,521,123
CG31	30,053,703	13,431,205	16,622,498
CG30	23,827,273	5,229,604	18,597,669
CG39	27,583,961	26,398,878	1,185,083

CG15	14,712,454	13,553,122	1,159,332
CG8	22,013,073	7,530,061	14,483,012
CG5	13,686,417	7,480,451	6,205,966
CG36	13,776,261	8,375,037	5,401,224
CG7	17,440,262	9,712,215	7,728,047
CG25	18,719,871	17,421,937	1,297,934
CG6	15,421,323	8,697,106	6,724,217
CG3	13,798,220	4,658,142	9,140,078
CG4	19,644,461	18,940,202	704,259
CG32	17,102,518	16,140,507	962,011
CG20	13,731,109	5,300,771	8,430,338
CG38	14,401,946	8,876,477	5,525,469
CG40	22,047,106	11,217,765	10,829,341
CG19	13,690,527	11,436,403	2,254,124
CG29	21,139,680	10,381,874	10,757,806
CG21	15,931,002	10,665,618	5,265,384
CG41	22,681,132	14,973,990	7,707,142
CG22	20,977,825	19,530,666	1,447,159
CG1	24,278,045	19,582,545	4,695,500
CG14	24,595,369	3,873,114	20,722,255
CG26	21,545,134	12,126,495	9,418,639
CG2	20,157,231	5,466,117	14,691,114
CG42	23,314,043	6,251,348	17,062,695
CG11	21,007,204	5,925,914	15,081,290
CG35	19,994,461	5,209,568	14,784,893
CG9	15,207,992	9,436,962	5,771,030
CG34	24,058,154	5,066,329	18,991,825
CG17	22,642,505	8,251,224	14,391,281
CG13	25,981,702	6,096,194	19,885,508
HG37	22,568,425	10,514,417	12,054,008
HG29	15,571,075	11,005,045	4,566,030
HG18	15,053,300	4,852,118	10,201,182
HG14	21,761,146	3,886,526	17,874,620
HG38	18,744,799	944,950	17,799,849
HG15	24,415,426	855,629	23,559,797
HG39	27,874,654	166,004	27,708,650
HG40	16,142,032	967,542	15,174,490
HG16	29,618,320	386,206	29,232,114
HG17	30,505,105	994,336	29,510,769
HG9	27,614,671	921,278	26,693,393
HG28	15,792,156	2,588,294	13,203,862
HG34	19,511,833	3,340,595	16,171,238
HG10	28,941,490	1,318,476	27,623,014
HG30	37,757,928	10,863,240	26,894,688

HG31	17,835,806	1,454,697	16,381,109
HG32	33,195,820	1,386,738	31,809,082
HG33	31,171,477	1,984,462	29,187,015
HG11	26,425,763	23,844,643	2,581,120
HG12	37,492,260	13,262,380	24,229,880
HG19	27,171,105	10,409,816	16,761,289
HG35	31,773,463	17,712,967	14,060,496
HG36	26,347,749	9,959,578	16,388,171
HG1	30,853,958	2,294,084	28,559,874
HG13	33,642,727	4,354,221	29,288,506
HG21	32,751,157	418,646	32,332,511
HG2	29,511,669	28,104,541	1,407,128
HG22	30,363,568	28,701,910	1,661,658
HG23	30,732,808	29,075,021	1,657,787
HG24	25,127,801	24,019,024	1,108,777
HG25	31,236,215	15,605,751	15,630,464
HG3	30,512,358	29,439,121	1,073,237
HG26	20,053,307	19,332,580	720,727
HG27	27,793,819	26,949,199	844,620
HG4	24,945,061	19,397,194	5,547,867
HG5	30,868,027	29,772,354	1,095,673
HG6	28,170,309	26,885,484	1,284,825
HG7	29,165,798	28,157,615	1,008,183
HG8	31,675,674	30,562,835	1,112,839
HG20	30,505,239	29,352,412	1,152,827
NC1	645,649	99,968	545,681
NC2	6,409,819	912,474	5,497,345
NC3	14,421,389	5,089,277	9,332,112
NC4	11,764,432	1,543,727	10,220,705
NC5	650,867	114,628	536,239
NC6	27,200,744	1,179,993	26,020,751

Table S5. The total number/abundance of the 534 genera of bacteria.

Table S6. Statistical analysis of antibiotic resistance gene abundance in figure 6a.

Antibiotic resistance genes	P-value (HCG VS PDG)
arlR	0.129372788
CpxR	0.04181358
gyrB	0.669322139
smeR	0.050672372
PIB	0.000724031
evgA	0.300596598

gyrA	0.655616773
rpld	0.724091892
ErmT	0.065789612
Erm(48)	0.065789612
Erm(44)v	0.065789612
ErmG	0.065789612
ErmC	0.065789612
ErmY	0.065789612
Erm(33)	0.162315334
Erm(43)	0.065789612
ErmA	0.162315334
ErmB	0.065789612
erm(45)	0.065789612
Erm(47)	0.065789612
PhoP	0.201514367
rpoB	0.167354089
mtrA	0.144795591
parY	0.617595665
kdpE	0.097127446
rpsL	0.993132404
dfrG	0.45937984
dfrK	0.068785742
dfrD	0.112476821
dfrC	0.416762539
dfrE	0.538803593
dfrA3	0.509810087
EF-Tu	0.118586518
parE	0.750404304
PtsI	0.209966759
murA	0.283632654
UhpA	0.573428205
fusA	0.129711697
fusE	0.972454498
vanRG	0.063015425
vanRC	0.098618774
vanRM	0.07744892
vanRD	0.076992291
vanRL	0.072737588
vanRI	0.158039428
vanRO	0.65186859
vanRB	0.083103895
vanRF	0.047298832
vanRA	0.065867504
vanRN	0.351865584

vanRE	0.091289849
liaR	0.513868514
basR	0.117455578
rpsA	0.005455287
tetB(P)	0.04991975
tetT	0.891616885
tet32	0.298106066
tet(44)	0.406354145
tetO	0.901994778
tetW	0.019900335
tetS	0.972970733
otr(A)	0.501865286
tet(W/N/W)	0.008387276
rpsJ	0.173034568
tetM	0.959515952
tet36	0.028115484
fabG	0.402633121

Table S7. Statistical analysis of antibiotic resistance gene abundance in figure 6b.

Antibiotic resistance genes	P-value (PDG VS CG)
arlR	0.00051016
CpxR	0.000504217
smeR	0.000158128
PIB	0.290223987
rpld	0.371881498
ErmT	1.10E-05
Erm(48)	1.10E-05
Erm(44)v	1.10E-05
ErmG	1.10E-05
ErmC	1.10E-05
ErmY	1.10E-05
Erm(33)	0.000104181
Erm(43)	1.10E-05
ErmA	0.000104181
ErmB	1.10E-05
erm(45)	1.10E-05
Erm(47)	1.10E-05
PhoP	4.12E-05
mtrA	5.73E-05
kdpE	0.01088132
rpsL	0.893311364
dfrK	0.00451831

dfrD	0.015530789
EF-Tu	0.670590848
PtsI	0.003391732
murA	0.02720898
fusA	0.289998824
fusE	0.593134459
vanRG	0.005407661
vanRC	0.000118639
vanRM	0.006901466
vanRD	0.005015205
vanRL	0.000114582
vanRI	0.00013626
vanRB	0.005544515
vanRF	0.005143066
vanRA	0.004417995
vanRN	0.001186038
vanRE	0.006433299
liaR	0.050272122
basR	0.0156464
tetB(P)	0.048860142
tetT	0.006400432
tet32	0.021448338
tet(44)	0.099692714
tetO	0.067659668
tetW	0.029046952
tetS	0.000468281
otr(A)	0.834523924
tet(W/N/W)	0.015931072
rpsJ	0.282888118
tetM	0.00126816
tet36	0.005943026
fabG	0.764754518
